# In Situ-Forming Collagen-Hyaluronate Semi-Interpenetrating Network Hydrogel Enhances Corneal Defect Repair

**DOI:** 10.1167/tvst.11.10.22

**Published:** 2022-10-14

**Authors:** Fang Chen, David C. Mundy, Peter Le, Youngyoon Amy Seo, Caitlin M. Logan, Gabriella Maria Fernandes-Cunha, Chris A. Basco, David Myung

**Affiliations:** 1 Department of Ophthalmology, Byers Eye Institute, Stanford University School of Medicine, Palo Alto, CA, USA; 2 VA Palo Alto HealthCare System, Palo Alto, CA, USA; 3 Department of Chemical Engineering, Stanford University, Palo Alto, CA, USA

**Keywords:** corneal substitute, tissue regeneration, keratoplasty, hydrogel, collagen, hyaluronic acid, semi-interpenetrating polymer network (SIPN)

## Abstract

**Purpose:**

Millions worldwide suffer vision impairment or blindness from corneal injury, and there remains an urgent need for a more effective and accessible way to treat corneal defects. We have designed and characterized an in situ-forming semi-interpenetrating polymer network (SIPN) hydrogel using biomaterials widely used in ophthalmology and medicine.

**Methods:**

The SIPN was formed by cross-linking collagen type I with bifunctional polyethylene glycol using N-hydroxysuccinimide ester chemistry in the presence of linear hyaluronic acid (HA). Gelation time and the mechanical, optical, swelling, and degradation properties of the SIPN were assessed. Cytocompatibility with human corneal epithelial cells and corneal stromal stem cells (CSSCs) was determined in vitro, as was the spatial distribution of encapsulated CSSCs within the SIPN. In vivo wound healing was evaluated by multimodal imaging in an anterior lamellar keratectomy injury model in rabbits, followed by immunohistochemical analysis of treated and untreated tissues.

**Results:**

The collagen-hyaluronate SIPN formed in situ without an external energy source and demonstrated mechanical and optical properties similar to the cornea. It was biocompatible with human corneal cells, enhancing CSSC viability when compared with collagen gel controls and preventing encapsulated CSSC sedimentation. In vivo application of the SIPN significantly reduced stromal defect size compared with controls after 7 days and promoted multilayered epithelial regeneration.

**Conclusions:**

This in situ-forming SIPN hydrogel may be a promising alternative to keratoplasty and represents a step toward expanding treatment options for patients suffering from corneal injury.

**Translational Relevance:**

We detail the synthesis and initial characterization of an SIPN hydrogel as a potential alternative to lamellar keratoplasty and a tunable platform for further development in corneal tissue engineering and therapeutic cell delivery.

## Introduction

An estimated 12.7 million people across the globe suffer from corneal blindness.^1^^,^^6^ Corneal diseases and injuries that penetrate the epithelium and affect the stroma are particularly likely to cause fibrotic scarring that threaten vision. The definitive treatment for corneal blindness is transplantation, which can be used to address not only long-standing opacities but also acute injuries, ulcers, and melts.^2^^,^^3^ Cyanoacrylate adhesives have been used off-label to seal corneal defects; however, they are only temporizing agents until surgery can be performed, can release toxic compounds like formaldehyde, and can induce inflammation and neovascularization.^4^^,^^5^ Owing to donor shortages and a lack of health care infrastructure, only 1 in 70 patients worldwide have access to cadaveric donor corneas,^1^^,^^6^ with the shortage of graft tissue projected to get even worse in years to come.^7^ Moreover, 6% of anterior lamellar grafts fail within 10 years,^8^ with up to 30% of grafts failing for certain indications.^9^

Several corneal replacement strategies have been advanced to overcome reliance on donor tissue. Preformed core-skirt keratoprostheses like the US Food and Drug Administration (FDA)–approved poly(methyl methacrylate)-based Boston Keratoprosthesis^10^ have helped patients with a poor transplant prognosis but are limited in clinical use.^11^^,^^12^ To better approximate the physiologic, viscoelastic properties of the cornea, decellularized porcine stroma implantation has successfully improved transparency,^13^^,^^14^ with current efforts aimed at improving decellularization methods and decreasing zoonotic risk.^15^ Moreover, biosynthetic hydrogel implants using recombinant human collagen type III have restored corneal integrity and improved vision in patients with keratoconus^16^^–^^18^ and ulceration.^19^

There remains a global need for transplant alternatives that can be used in settings without access to surgery. Additional challenges include the difficulty of sizing and handling implants and complications such as neovascularization secondary to suturing.^20^ Recent efforts have been aimed at developing in situ-forming corneal substitutes.^21^^,^^22^ Collagen type I is a particularly promising biomaterial for corneal regeneration since it is the most abundant protein in the native stromal extracellular matrix (ECM).^23^ Our laboratory has recently developed in situ-forming hydrogels composed of collagen type I and bioinert polyethylene glycol (PEG) using N-hydroxysuccinimide (NHS) ester chemistry that improved wound healing and reduced scar formation in rabbits with lamellar injury.^24^ Hyaluronic acid (HA), or hyaluronan, is a glycosaminoglycan constituent of the ECM expressed in the cornea^25^ and is upregulated during injury.^26^ HA is widely used to treat dry eye^27^ and in periocular fillers^28^ and has been shown to promote re-epithelialization,^29^^,^^30^ decrease inflammation^31^ and scar formation,^32^^,^^33^ and promote healing after corneal injury.^34^

We have previously shown that incorporating HA into a bio-orthogonally cross-linked collagen hydrogel improved mechanical properties compared with collagen alone^35^ and that an HA–collagen simultaneous interpenetrating polymer network facilitated suture-free defect repair in vivo.^36^ Here we report a novel, in situ-forming semi-interpenetrating polymer network (SIPN) composed of HA and collagen, which combines the biomechanical properties of the two constituents and simulates the composition of native corneal matrix. An SIPN is defined as a polymer entangled within but not covalently crosslinked to another polymer network.^37^ In our case, HA is dispersed within a collagen network that is cross-linked with PEG via NHS ester chemistry. PEG is biocompatible and used, for instance, in numerous therapeutics, including the FDA-approved ophthalmic insert DEXTENZA (Ocular Therapeutix, Bedford, MA). Moreover, NHS ester chemistry is applied in ReSure (Ocular Therapeutix), the only FDA-approved PEG-based sealant for corneal incisions.^38^ The in situ-forming SIPN hydrogel stromal replacement described in this study can form in situ and supports surface epithelialization, greatly expanding the scope of potential clinical application and obviating the need for sutures, photoinitiators, and lengthy photocross-linking that require significant surgical infrastructure. Here, we investigate the SIPN's biomechanical and optical properties, as well as its cytocompatibility and ability to enhance in vivo wound healing, with the goal of developing an alternative to current methods for treating corneal wounds and opacities.

## Methods

### Synthesis of Hydrogels

Type I bovine collagen (ThermoFisher Scientific, Waltham, MA; A1064401) was first pH neutralized using a solution of 1.0 M sodium hydroxide solution, deionized water, and 10× phosphate-buffered saline (PBS) in a 3:57:20 ratio. The 5 mg/mL collagen solution was mixed with neutralization solution to a concentration of 3 mg/mL. Neutralized collagen was cross-linked with succinimidyl glutamic acid ester bifunctionalized PEG 2k (Creative PEGworks, Durham, NC; PSB-3312) using NHS ester chemistry to react with collagen's primary amines. First, PEG was solubilized in PBS to a concentration of 100 mg/mL, then 3.2 µL of this solution was added to 100 µL of neutralized collagen. To form the SIPN, HA (Sigma-Aldrich, St Louis, MO; 42686) was solubilized in PBS to a concentration of 25, 50, or 100 mg/mL, then 3 µL was added to the cross-linked collagen. To form the conjugated HA-Col gel, neutralized collagen was conjugated to NHS ester functionalized HA (Creative PEGworks; HA-342) via NHS chemistry before PEG cross-linking. First, HA-NHS was solubilized in PBS to a concentration of 50 mg/mL, then 3 µL of this solution was added to 100 µL of neutralized collagen, followed by PEG cross-linking as described elsewhere in this article. For the noncovalently cross-linked collagen hydrogel, a physically cross-linked gel commonly used in cell culture, collagen was neutralized as described elsewhere in this article. Solutions were placed at 37°C for 30 minutes for gelation.

### Reaction Efficiency of Hydrogels

Collagen conjugation efficiency was determined by fluorescamine assay. Each hydrogel (containing 50 µL of neutralized collagen) was mixed with 25 µL of 3 mg/mL fluorescamine (Sigma-Aldrich; F9015) in DMSO. After a 70-minute incubation at room temperature with foil wrap to prevent light exposure, fluorescence intensity (Ex/Em = 380/470 nm) was measured using a SpectraMax M Series Multi-Mode Microplate Reader (Sunnyvale, CA). Using the linear standard curve generated by neutralized collagen from 0 to 3 mg/mL, conjugation efficiency was evaluated.

### Mechanical Characterization of Hydrogels

The rheological properties of the gels were measured using an ARES-G2 rheometer (TA Instruments, New Castle, DE) with a 25-mm parallel plate. Hydrogels were synthesized in situ. Time sweeps were measured under 1% strain and 1 Hz oscillatory frequency at 37°C until storage modulus plateau. Frequency sweeps were performed under 1% strain with an oscillatory frequency from 0.1 to 10.0 Hz.

### Optical Characterization of Hydrogels

To evaluate transparency, 100 µL of hydrogels were formed in a 96-well plate, and the absorbance was measured between 380 and 700 nm (Tecan Microplate Reader). PBS was used as the blank. Transmittance was calculated using the relationship T (%) = 1/10^A^ × 100, where A is the absorbance. The refractive indices of the hydrogels were measured with a digital refractometer (HI96800, Hanna Instruments, Woonsocket, RI). The machine was calibrated with double distilled water.

### Swelling and Degradation of Hydrogels

In vitro degradation of hydrogels was performed in the presence of 10 U/mL of collagenase (Sigma-Aldrich; C0130) or 10 U/mL of hyaluronidase (Sigma-Aldrich; H3631) in PBS at 37°C on an orbital shaker.^39^ Gels soaked in PBS were used as a negative control. Hydrogels (200 µL each) were preswollen in PBS (pH 7.4) overnight. At desired time points, the gels were removed from the degradation buffer, blotted with Kimwipes (Kimberly-Clark Professional, Irving, TX), dried for 1 hour under ambient conditions (20°C, 40% humidity), and weighed. The percent of degraded gels was determined by the following formula: degraded (%) = 100 × (1 – W_t_/W_0_), where W_0_ is the initial weight, and W_t_ is the weight at time t.

For the in vitro swelling of hydrogels, 200 µL of hydrogels were placed in PBS, and the weights of each hydrogel were recorded at varying time points. Swelling was calculated using the following equation: swelling (%) = (W_t_ – W_0_)/W_0_ × 100, where W_0_ is the initial weight, and W_t_ is the weight at time t.

### Corneal Cell Culture

The human immortalized corneal epithelial cells (ICECs) is from a cell line (CRL-11135, human corneal epithelium [HCE-2]) purchased from ATCC (Manassas, VA, USA). They were cultured^24^ in keratinocyte serum-free media (ThermoFisher Scientific) supplemented with bovine pituitary extract 0.05 mg/mL (ThermoFisher Scientific), human recombinant epithelial growth factor 5 ng/mL (ThermoFisher Scientific), hydrocortisone 100 ng/mL (Sigma-Aldrich), insulin 5 µg/mL (Sigma-Aldrich), and 1% antibiotic antimycotic solution (Sigma-Aldrich).

Corneal stromal stem cells (CSSCs) were harvested from human donor corneas provided by Lions Eye Institute (Slingerlands, NY) as previously described.^24^ CSSCs were cultured in Minimum Essential Medium Eagle (Sigma-Aldrich; M4526) containing 10% fetal bovine serum, 1% antibiotic antimycotic solution (Sigma-Aldrich; A5955), 1% nonessential amino acid solution (Sigma-Aldrich; M7145) and 1% Glutamax (ThermoFisher Scientific; 35,050,061).

### Cytocompatibility of Hydrogels

Corneal cell viability was assessed using the calcein AM/ethidium homodimer-1 LIVE/DEAD assay (ThermoFisher Scientific). For the ICECs, 200 µL hydrogels were formed in a 12-well plate, then 150,000 cells were seeded on the gels with 200 µL of medium. For the CSSCs, 100,000 cells were suspended in 200 µL hydrogels before gelation, then gels were formed in an 8-well chamber slide, and 200 µL of medium was added. After 48 hours, cells were incubated with calcein AM (1:1000) and ethidium homodimer-1 (1:500) in media for 45 minutes then imaged (Keyence BZ-X810). Viability was assessed using CellProfiler cell image analysis software.

### CSSC Distribution in Hydrogels

The distribution of CSSCs was evaluated by staining cells with Alexa Fluor 488 Phalloidin (ThermoFisher Scientific; A12379). First, 80,000 cells were encapsulated in 200 µL hydrogels before gelation, then gels were formed in an 8-well chamber slide, and 200 µL of medium was added. At 24 and 96 hours, the cells were fixed with 4% paraformaldehyde for 15 minutes, then permeabilized and blocked with 0.5% triton-X and 5% goat serum in PBS for 30 minutes. Alexa Fluor 488 Phalloidin was then added to the cells in PBS (1:40) for 30 minutes. The cells were imaged using fluorescence microscopy (Keyence BZ-X810). Briefly, z-stack images of at least three samples were acquired at 50-µm intervals from the bottom to the top of hydrogels. Quantitative analysis of the images was performed using ImageJ (version 1.53; NIH, Bethesda, MD). To visualize relative distribution, cell count at each level was normalized to the total cell count of that z-stack.

### In Vivo Lamellar Keratectomy Studies

Adult New Zealand white rabbits were used in this study. Animal experiments were designed to conform with the ARVO Statement for the Use of Animals in Ophthalmic and Vision Research and were reviewed and approved by the Stanford University Institutional Animal Care and Use Committee. All anesthesia techniques were performed by the veterinary service center at Stanford University. Animals were randomly divided into three groups: SIPN (*n* = 3), the collagen gel (*n* = 3), and PBS control (*n* = 2). Before surgery, one drop of proparacaine hydrochloride ophthalmic solution was added to the eye receiving treatment. Central corneal thickness was measured by pachymetry (Handy Pachymeter SP-100, Tomey, AZ). Lamellar keratectomy was performed on the right eye using a 3.5-mm inner-stopper guarded trephine^40^ with a depth of 260 µm calibrated between the blade and inner stopper to create a standardized circular cut and a spatula to remove the collagen fibril layers. We added 5 µL of premixed hydrogel or PBS into the wounded site, allowed it to sit for 5 minutes, and covered it with a contact lens to prevent scratching. A tarsorrhaphy was then performed to prevent agitation by the animal and help keep the contact lens and gel in place. Ofloxacin ophthalmic solution was applied daily. On day 7, the tarsorrhaphy and contact lens were removed for eye examination. Examination on days 0 and 7 included photographs captured with a digital portable slit lamp (LED16, MicroClear Medical).^41^ Optical coherence tomography (OCT) of the anterior eye segment was conducted using the SPECTRALIS Heidelberg Retina Angiograph + OCT (Heidelberg Engineering Inc., Heidelberg, Germany). OCT images were analyzed with ImageJ (version 1.53, NIH). To evaluate the lamellar keratectomies, the percentage of corneal thickness excised was calculated using the formula: 100 × (thickness of intact cornea adjacent to keratectomy – thickness of residual stromal bed at keratectomy)/thickness of intact cornea adjacent to keratectomy. Absolute cut depth was obtained by multiplying excised fraction by the presurgical central corneal thickness. To assess stromal wound healing at the keratectomy site, the defect percentage was calculated using the formula: 100 × (defect area/total area including residual stromal bed). To assess epithelial wound healing, corneas were stained with saline-moistened fluorescein dye strips (Haag-Streit, Koeniz, Switzerland) and imaged under cobalt blue illumination. Corneal opacity was quantified from magnified photos using a modified McDonald–Shadduck scoring system from 0 to 4.^42^ On day 7, the rabbits’ eyes were enucleated and fixed in 4% paraformaldehyde, with the left contralateral eyes serving as unoperated controls. The fixed cornea tissues were embedded in Tissue-Tek OCT compound and then cryosectioned for immunohistochemical analysis.

### Immunohistochemical Analysis

The fixed samples were washed with PBS thrice and then incubated overnight with primary antibodies to alpha-smooth muscle actin (α-SMA) or zonula occludens-1 in 0.5% triton-x and 5% normal goat serum. Next, the secondary antibody anti-mouse Alexa 546 was added. After washing, the sections were incubated with DAPI for 5 minutes. The sections were then mounted and imaged with a confocal microscope (Leica TCS SP5).

### Statistical Analyses

Each experiment was repeated at least three times unless otherwise indicated, and data are expressed as the mean ± standard deviation (^#^*P* < 0.05, ^##^*P* < 0.01, ^###^*P* < 0.001, and ^####^*P* < 0.0001). One-way or two-way analysis of variance was performed, followed by Tukey's or Šídák's multiple comparisons test for statistical analysis as indicated (GraphPad Prism 9.1.2, GraphPad Software, San Diego, CA). For CSSC distribution analysis, the Stoner-Kim robust statistical method was used to compare day 1 and day 4 distributions at each height. Confidence intervals were determined using the Kulinskaya–Morgenthaler–Staudte method, and critical *P* values were determined using Hochberg's multiple comparison adjustment. The earth mover's distance (EMD) was computed using the ‘emdist’ package (R version 3.4.4).

## Results

### Mechanical Properties of Hydrogels

**Figure 1.**
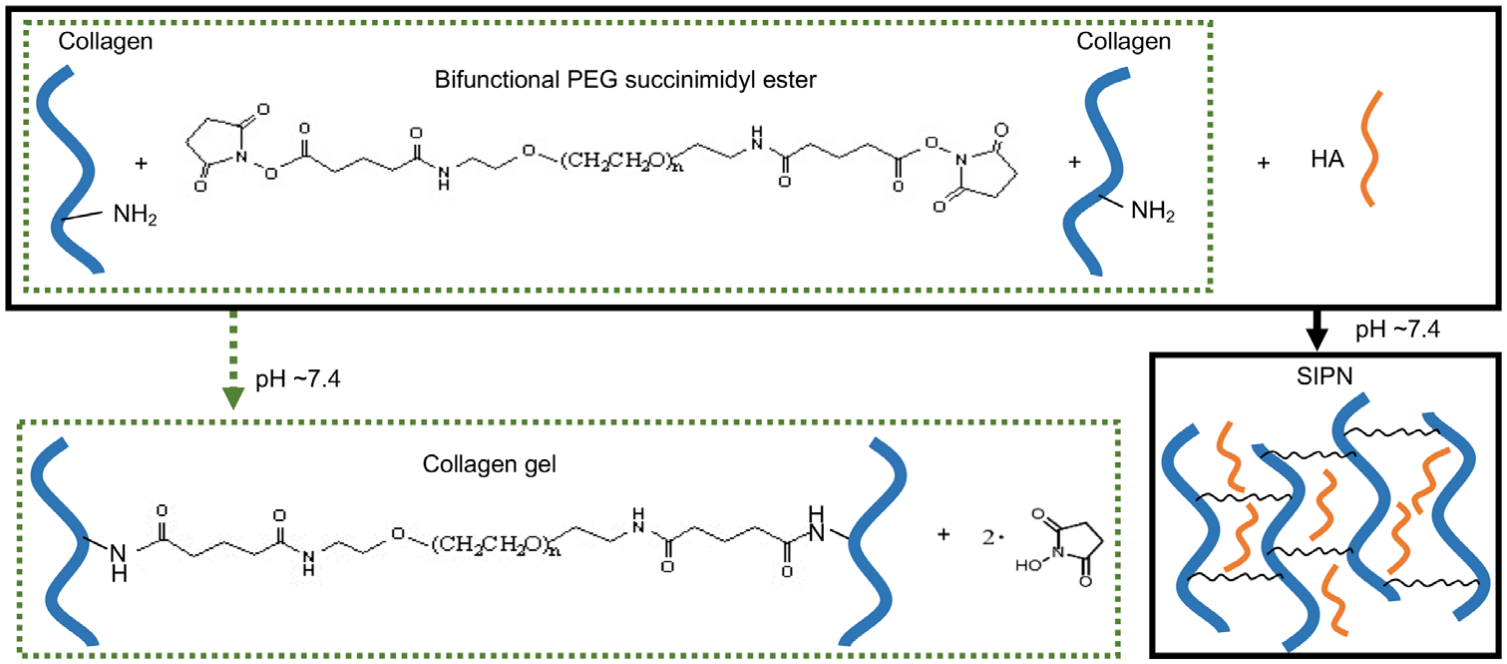
Schematic of hydrogel formation under physiologic conditions. The collagen gel was formed via cross-linking between primary amines on collagen and NHS groups on bifunctionalized PEG. The SIPN was formed by entangling HA in the crosslinked collagen.

**Figure 2.**
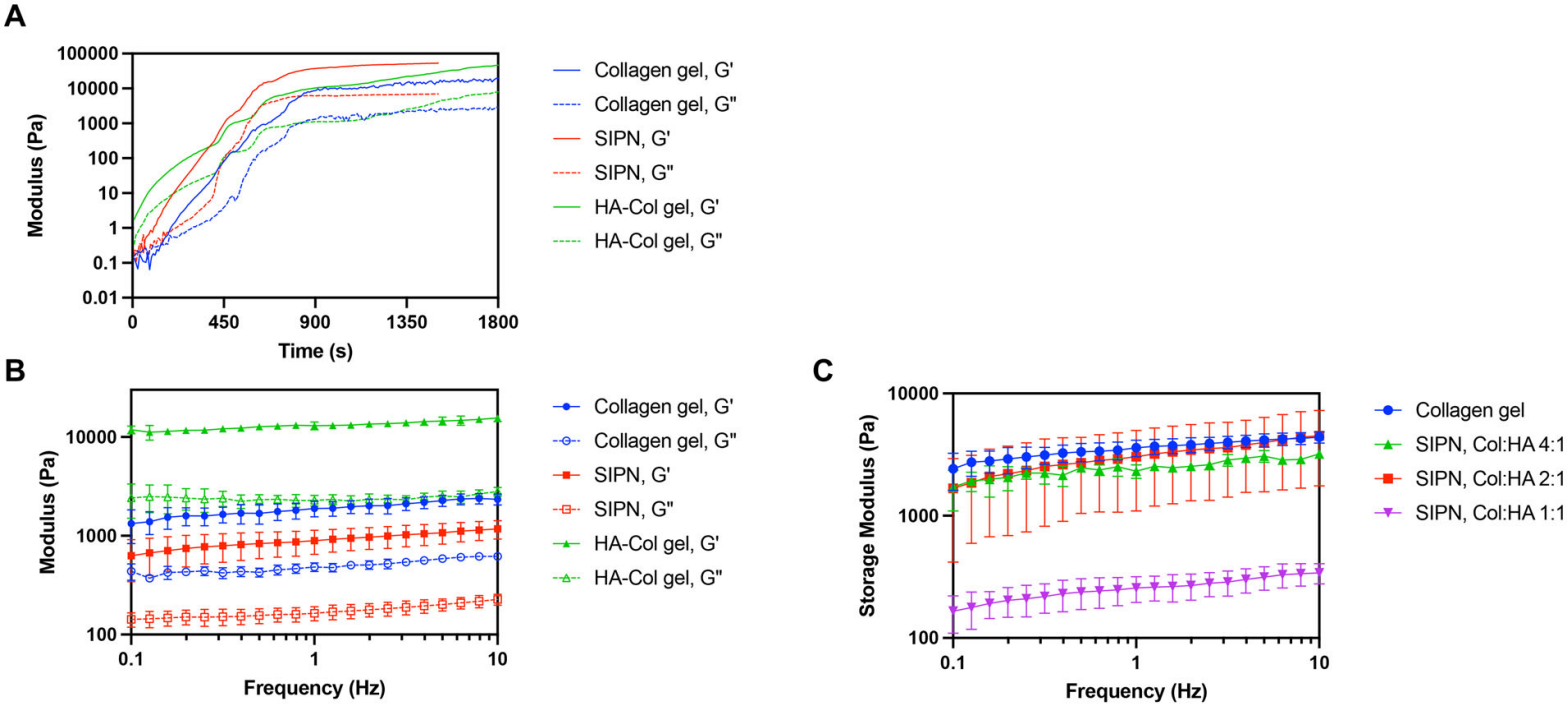
Mechanical properties of in situ-forming hydrogels. (**A**) Dynamic moduli of the SIPN (with physically entangled HA) compared with collagen and cross-linked HA-collagen (HA-Col) gels as a function of time during gelation. (**B**) Dynamic moduli of gels as a function of frequency. Gʹ is storage modulus and Gʹʹ is loss modulus. At 10 Hz, storage modulus of HA-Col was significantly greater than collagen gel and SIPN (*P* < 0.0001). (**C**) Storage modulus of SIPN at different concentrations of HA (Col:HA 4:1, Col:HA 2:1, Col:HA 1:1 w/w) as a function of frequency. At 10 Hz, storage moduli of the collagen gel and SIPN, Col:HA 2:1 were significantly greater than the SIPN, Col:HA 1:1 (*P* < 0.05). Data are reported as mean ± standard deviation and compared using an ordinary one-way analysis of variance followed by Tukey's multiple comparisons test.

To create a corneal matrix substitute for facile clinical use to optimize wound healing, the properties of collagen and HA were combined in an SIPN hydrogel by first using NHS ester chemistry to cross-link collagen with bifunctionalized PEG (collagen gel) and then adding HA (Fig. 1). Collagen was conjugated to PEG with a mean efficiency of 39% after 30 minutes by fluorescamine assay, which decreased to 24% with the addition of HA to form the SIPN, likely owing to steric effects. The mechanical properties of the hydrogels were measured using oscillatory rheology (Fig. 2). To additionally understand how the method of HA incorporation affects hydrogel properties, a collagen-HA (HA-Col) gel are synthesized where HA was chemically cross-linked to collagen. The SIPN and cross-linked HA hydrogels began to form immediately, and the collagen gel within minutes, indicated by the point at which the storage modulus (Gʹ) became greater than loss modulus (Gʹʹ), an estimate of initial gelation (Fig. 2A).^43^ The dynamic moduli of all gels reached one-half maximum, representing 50% gelation, within 10 minutes. The SIPN gel reached its equilibrium modulus the fastest at approximately 13 minutes, followed by the collagen gel at approximately 15 minutes and HA-Col gel at approximately 30 minutes. To further characterize the hydrogels, gels were formed for 30 minutes, and Gʹ and Gʹʹ were measured as a function of frequency from 0.1 to 10.0 Hz (Figs. 2B, C). Complete gelation was confirmed since the loss tangent (tan δ = Gʹʹ/Gʹ) was frequency independent for all hydrogels by F-test for linear regression. The SIPN reached a storage modulus of 1.17 ± 0.25 kPa, lower but not significantly different from the collagen gel (Fig. 2B). Incorporating HA through chemical conjugation significantly increased stiffness compared with the collagen gel and SIPN, with the HA-Col gel demonstrating a storage modulus of 15.63 ± 1.28 kPa (*P* < 0.0001). To determine whether the mechanical properties of the SIPN could be tuned by HA content, gels with different concentrations of HA (Col:HA 4:1, Col:HA 2:1, Col:HA 1:1 w/w) were assessed (Fig. 2C). The baseline SIPN (Col:HA 2:1) demonstrated the highest storage modulus, affirming the use of this concentration for subsequent experiments. Decreasing HA content (Col:HA 4:1) resulted in a slightly reduced storage modulus, whereas increasing HA content (Col:HA 1:1) significantly reduced storage modulus compared with both the collagen gel and baseline SIPN (Col:HA 2:1) (*P* < 0.05).

### Optical Properties of Hydrogels

**Figure 3.**
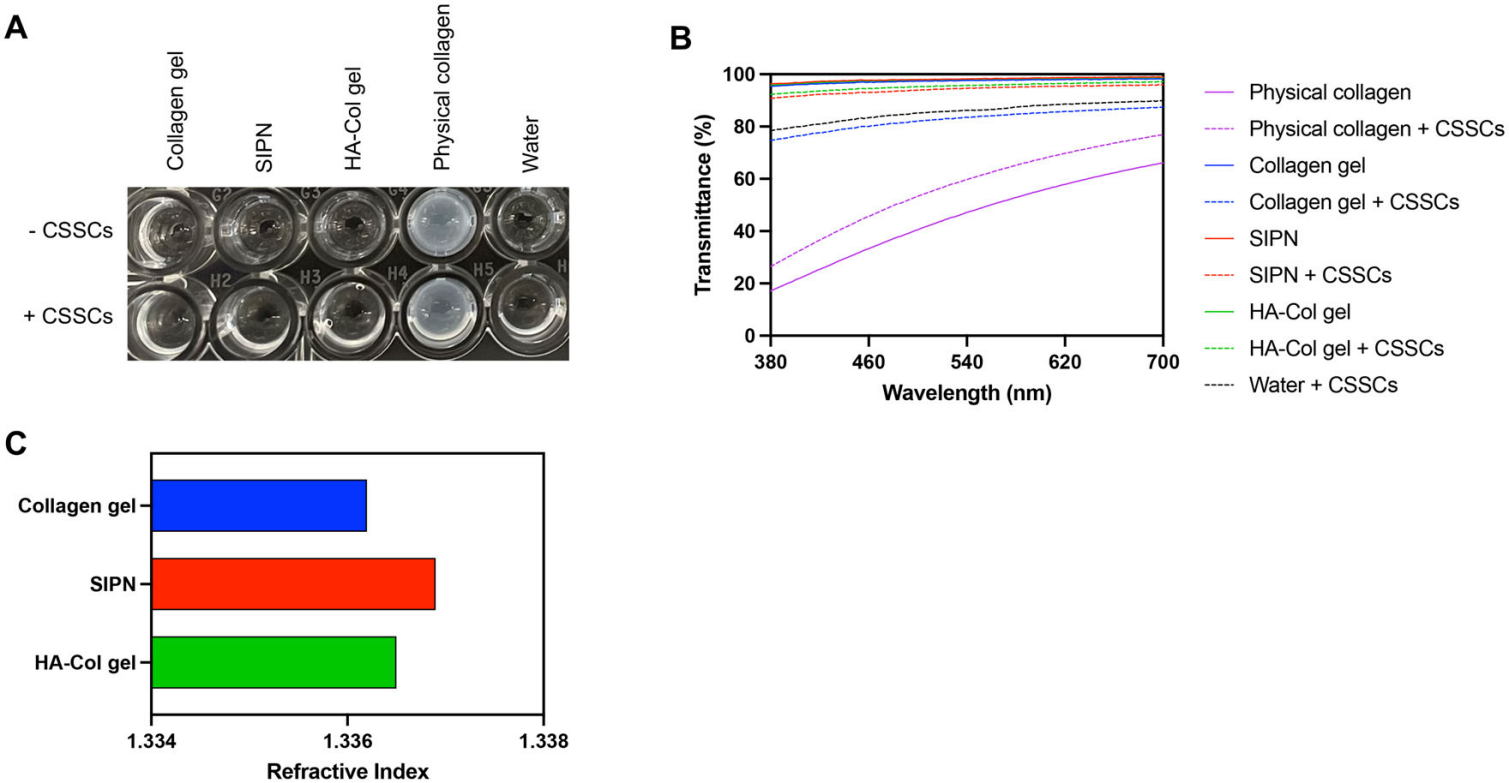
Optical properties of hydrogels. (**A**) Photograph of hydrogels with and without encapsulated CSSCs. Physical collagen and water are included for comparison. (**B**) Transmittance of hydrogels in the visible spectrum with and without encapsulated CSSCs. (**C**) Refractive index of hydrogels.

For optimal functional integration as a corneal defect filler, it is important that the SIPN have optical properties consistent with the native cornea. The optical properties of the hydrogels were assessed both alone and with encapsulated CSSCs to more closely simulate the native cornea (Fig. 3). Compared with nonchemically cross-linked physical collagen, the SIPN, collagen gel, and HA-Col gels were substantially more transparent with and without CSSCs, exhibiting a transparency similar to water (Fig. 3A). Transparency was quantified by measuring hydrogel transmittance in the visible light spectrum at wavelengths between 380 and 700 nm (Fig. 3B). The SIPN, collagen gel, and HA-Col gels demonstrated more than 95% transmittance in the visible range, with the SIPN showing the highest mean transmittance (98.12 ± 0.69%). These values meet the standard of the human cornea, with transmittance increasing from 80% to nearly 95% over the same spectral range.^44^ Physical collagen had a substantially lower transmittance, ranging from approximately 17% to 49% with increasing wavelength. In the presence of CSSCs, all chemically cross-linked hydrogels experienced a decreased transmittance. However, although the mean transmittance decreased by 15% for the collagen gel, the addition of HA attenuated this change, with the SIPN decreasing by approximately 4% and the HA-Col gel by 2%. Aside from transparency, the refractive index is integral to visual function during healing, given that it is a major determinant of the optical power of the cornea.^45^ The mean refractive index of the human cornea is approximately 1.376, with a slightly lower stromal refractive index (1.371).^46^^,^^47^ Of the gels tested, the SIPN had the refractive index closest to the human cornea at 1.337, with both gels that incorporate HA demonstrating higher refractive indices than the collagen gel (Fig. 3C).

### Swelling and Degradability of Hydrogels

**Figure 4.**
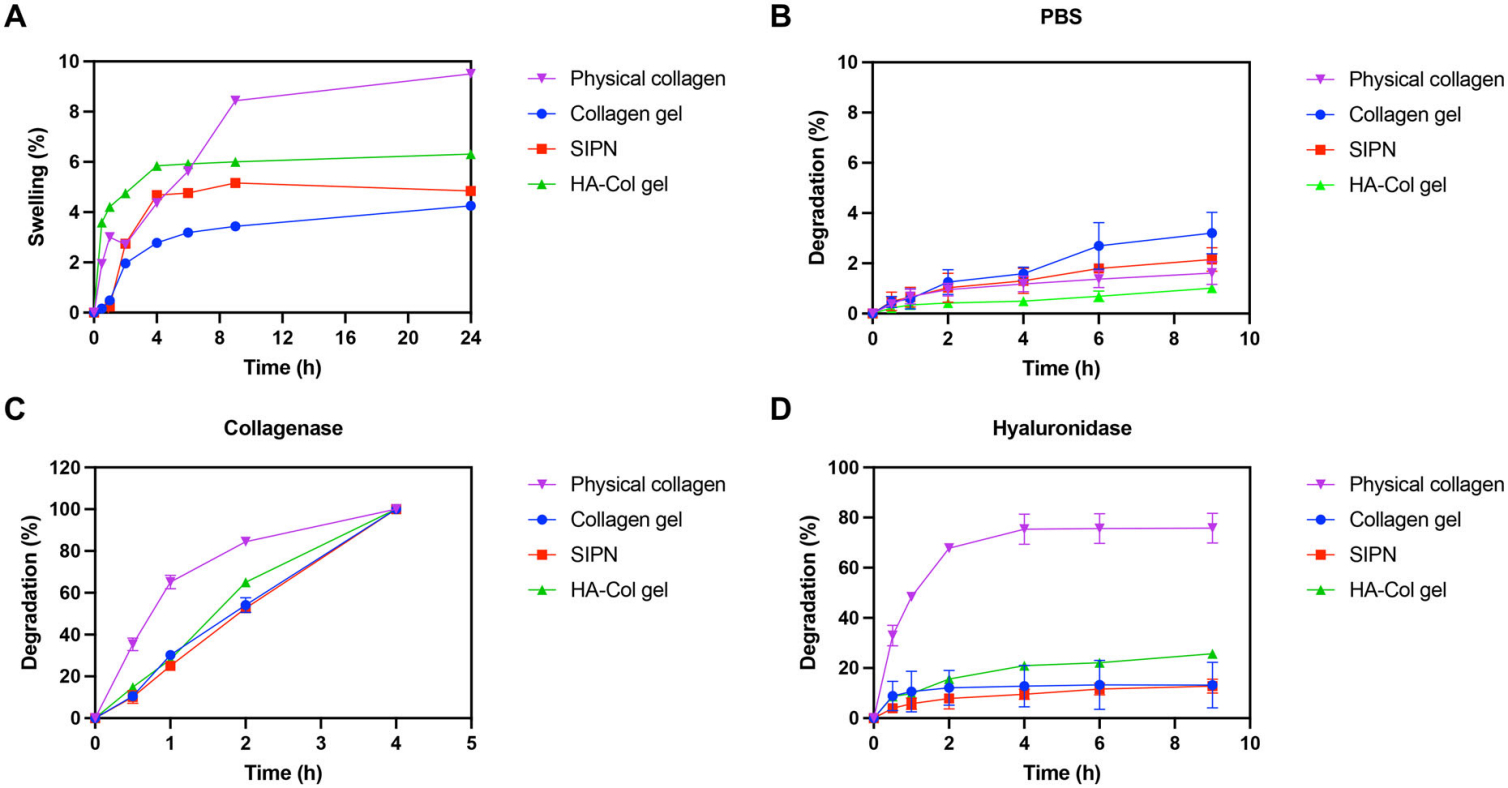
Swelling and degradation of the in situ-forming hydrogels. (**A**) Swelling of hydrogels in PBS over time. (**B**–**D**) In vitro degradation of hydrogels in the presence of PBS, collagenase, or hyaluronidase over time. (**B**) In the presence of PBS, all gels show 3% or less degradation at 9 hours. (**C**) In the presence of collagenase, physical collagen degraded significantly more than the collagen gel, SIPN, and HA-Col gel at 2 hours (*P* < 0.0001). The HA-Col gel degraded significantly more than the collagen gel and SIPN (*P* < 0.001). (**D**) In the presence of hyaluronidase, physical collagen degraded significantly more than the collagen gel, SIPN, and HA-Col gel at 9 hours (*P* < 0.0001). Data are reported as mean ± standard deviation and compared using an ordinary one-way analysis of variance followed by Tukey's multiple comparisons test.

Next, hydrogel swelling and degradability were characterized (Fig. 4). In the native cornea, symptomatic corneal edema occurs with swelling greater than 5% above its physiologic level, which can lead to separation of stromal lamellae, loss of transparency, and increased light scattering.^48^ Hydrogel swelling stability is important to preserve optimal transparency and topography.^49^ To explore these dynamics, the hydrogels were immersed in PBS, and swelling was measured over time (Fig. 4A). The swelling of the cross-linked hydrogels reached a plateau at approximately 4 hours. At 24 hours, the SIPN and collagen gel exhibited less than 5% swelling, while the HA-Col gel swelled around 6%. In contrast, the water content in physical collagen was greater than in all cross-linked gels by 6 hours and continued to increase until 24 hours.

Understanding of degradation behavior is critical for corneal tissue substitutes, as hydrogels should provide support and facilitate regeneration while also being gradually degraded and replaced by the healing cornea. During wound healing, collagenases and hyaluronidases are upregulated in the cornea to facilitate tissue remodeling and repair.^50^^,^^51^ Hydrogel degradation dynamics were assessed over time with PBS, 10 U/mL collagenase, or 10 U/mL hyaluronidase (Figs. 4B–D). In the presence of PBS, there was negligible mean degradation (≤3%) for all hydrogels, with no significant difference between gels at 9 hours (Fig. 4B). In the presence of collagenase, all gels were fully degraded by 4 hours (Fig. 4C). At 2 hours, physical collagen was degraded significantly more than all cross-linked hydrogels (*P* < 0.0001). Among the cross-linked gels, the HA-Col gel was degraded significantly more than the collagen gel and SIPN (*P* < 0.001), with the SIPN undergoing the least amount of degradation (52.6 ± 0.7%). In the presence of hyaluronidase, physical collagen was degraded significantly more than the cross-linked gels at 9 hours (*P* < 0.0001), and the SIPN was degraded the least (12.8 ± 2.8%) (Fig. 4D). Taken together, these results indicate that the SIPN, comparable to the other cross-linked hydrogels, demonstrates swelling stability and degrades more gradually compared with physical collagen.

### Hydrogel Cytocompatibility and Cell Distribution

**Figure 5.**
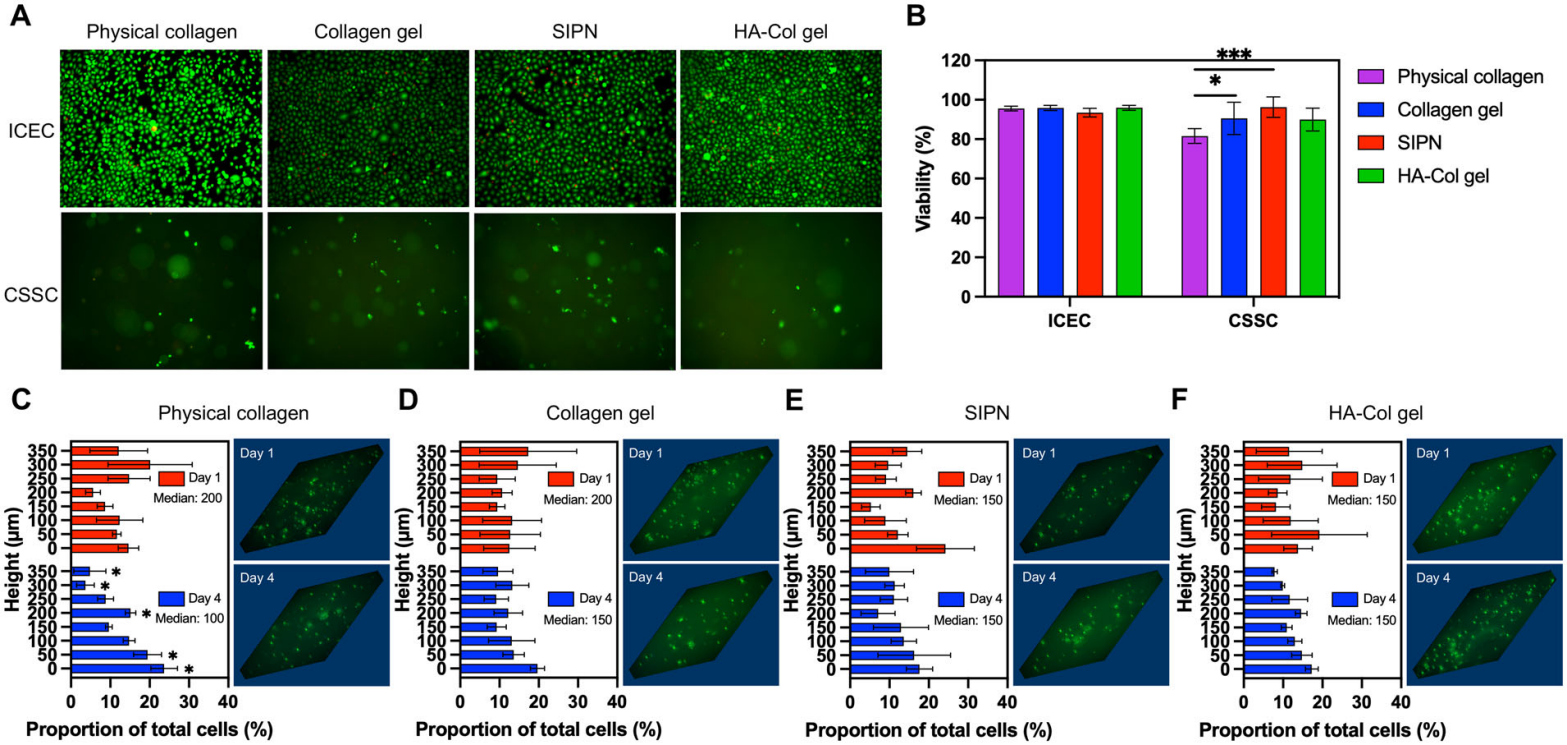
Cytocompatibility and cell distribution of in situ-forming hydrogels. (**A**) Representative live/dead images of ICECs seeded on and CSSCs encapsulated in hydrogels after 2 days in culture. Live cells are green and dead cells are red. (**B**) Quantification of corneal cell viability in hydrogels after 2 days in culture. Data are reported as mean ± standard deviation and compared using an ordinary one-way analysis of variance followed by Tukey's multiple comparisons test (*P < 0.05, ***P < 0.001). (**C**–**F**) Quantification and representative three-dimensional images of CSSC vertical distribution within (**C**) physical collagen, (**D**) collagen gel, (**E**) SIPN, and (**F**) HA-Col gel at 1 and 4 days after encapsulation. The height representing the median of the cell distribution at days 1 and 4 is indicated. Data are reported as mean ± standard deviation and distributions at day 1 and day 4 were compared at each height using the Stoner-Kim method followed by Hochberg's multiple comparison adjustment (*P < 0.05).

To further explore the ability of the SIPN to provide an effective stromal substitute, the hydrogels’ biocompatibility and influence on encapsulated CSSC distribution were evaluated (Fig. 5). The LIVE/DEAD assay was used to assess the viability of human ICECs seeded on the physical collagen and cross-linked hydrogels, as well as CSSCs encapsulated in the hydrogels (Fig. 5A). All gels were cytocompatible with ICECs, with more than 90% viability after 2 days (Fig. 5B). CSSC viability was more than 90% in all cross-linked gels, with the collagen gel maintaining significantly greater viability (90.6 ± 8.2%) than physical collagen at 81.6 ± 3.8% (*P* < 0.05). Notably, the SIPN with physically entangled HA significantly increased viability (96.2 ± 5.2%) compared with physical collagen (*P* < 0.001), whereas the cross-linked HA-Col gel (90.0 ± 5.8%) failed to augment viability significantly.

The spatial distribution of cells influences their morphology, proliferation, and function.^52^ Furthermore, the ability of hydrogels to resist gravity-mediated cell sedimentation before therapeutic administration is desirable for practical use in the clinic. For these reasons, the vertical distribution of encapsulated CSSCs was assessed via filamentous-actin staining and analysis of Z-stack images from the bottom to the top of the hydrogels acquired 1 and 4 days after encapsulation (Figs. 5C–F). The physical collagen gel was unable to maintain cell distribution from day 1 to day 4, with a downward shift in the median height level from 200 to 100 µm (Fig. 5C). There was a significant decrease in the proportion of cells in the upper one-quarter of the gel and a significant increase at the bottom quarter of the gel (*P* < 0.05). The cross-linked collagen gel exhibited a smaller shift in median height level from 200 to 150 µm, whereas the SIPN and HA-Col gels demonstrated no change (Figs. 5D–F). To further understand the ability of the hydrogels to preserve encapsulated cell distribution over time, the EMD, also known as the Wasserstein metric, was calculated between day 1 and day 4 for each gel. The EMD is an intuitive cross-bin metric to quantify the distance between probability distributions and can be thought of as the minimum cost of turning one cell distribution into the other, with the cost being the product of the cell proportion and distance moved.^53^^,^^54^ A larger EMD indicates greater change. As expected, physical collagen had the largest EMD (63.11) followed by the collagen gel (28.52), whereas the hydrogels incorporating HA showed a much smaller change in distribution from day 1 to day 4 (SIPN, 14.72; HA-Col, 14.33).

### SIPN Hydrogel Enhances Corneal Stromal Restoration

**Figure 6.**
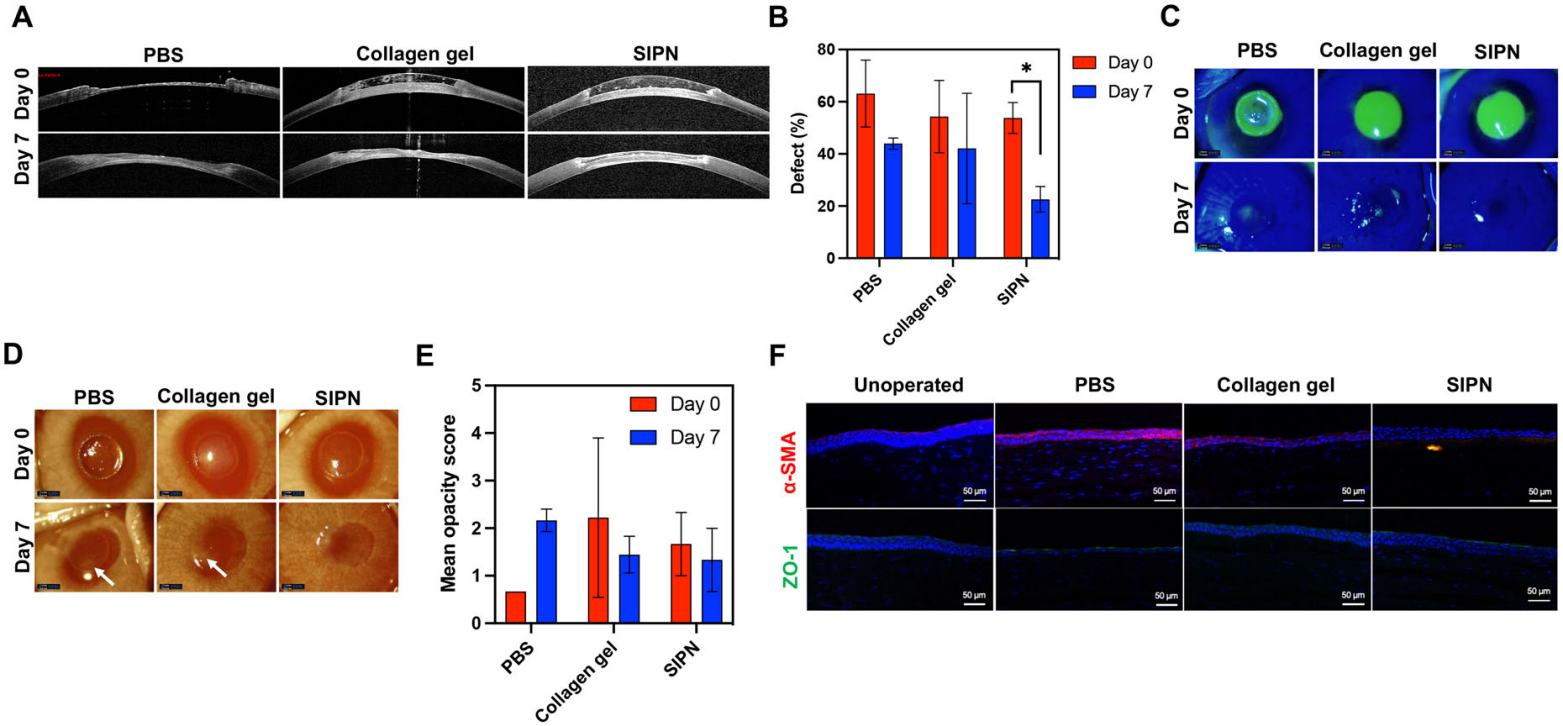
In vivo evaluation of the SIPN as a corneal stromal replacement. (**A**) OCT images of corneal defects treated with PBS, collagen gel, or SIPN at days 0 and 7. (**B**) Quantification of stromal defect sizes at days 1 and 7. The SIPN significantly reduced stromal defect size at 7 days (*P < 0.05). (**C**) Representative cobalt blue photos of fluorescein-stained corneas at days 0 and 7 demonstrating re-epithelialization. (**D**) Photos of treated eyes. White arrows indicate areas of opacity. (**E**) Quantification of corneal opacity using a modified McDonald-Shadduck scoring system from 0 to 4. Data are reported as mean ± standard deviation and compared using a two-way analysis of variance followed by Šídák's multiple comparisons test (*P < 0.05). (**F**) Immunofluorescence of treated corneas at 7 days after keratectomy. The top row was stained for α-SMA (*red*) and the bottom row was stained for zonula occludens-1 (ZO-1) (*green*). Cell nuclei were stained with DAPI (*blue*).

To assess the SIPN's effect on corneal wound healing in vivo, anterior lamellar keratectomies were performed in rabbits using a modified 3.5-mm inner-stopper guarded trephine^40^ to produce a mean wound depth of 264.0 ± 48.1 µm, or 68.2 ± 10.1% of the central corneal thickness, with no significant differences between treatment groups by ordinary one-way analysis of variance. PBS, the collagen gel, or SIPN was applied to injured corneas, and OCT demonstrated that both hydrogels fully filled the wounds and provided contiguous corneal curvature upon application (Fig. 6A). After 7 days, SIPN treatment significantly decreased the stromal defect size (based on central cross-sectional area) by 31.2 ± 3.8% of the central corneal thickness (*P* < 0.05), whereas PBS and collagen gel treatment failed to decrease the defect size significantly (Fig. 6B). Cobalt blue imaging of fluorescein-stained epithelial defects demonstrated re-epithelialization in all treatment groups at day 7, with the SIPN enhancing closure compared with the collagen gel (Fig. 6C). Because maintaining corneal transparency is crucial to functional biointegration, corneal opacity was assessed at days 0 and 7 after keratectomy (Fig. 6D). After 7 days, collagen gel-treated and SIPN-treated corneas were less opaque than PBS-treated corneas (Fig. 6E). Moreover, whereas PBS-treated corneas became more opaque over time, collagen gel and SIPN treatment decreased opacity, although this difference did not reach statistical significance. To further probe epithelial and stromal regeneration, animals were sacrificed at day 7, and corneal sections were analyzed by immunohistochemistry (Fig. 6F). SIPN treatment consistently supported multilayer (3–4 layers) re-epithelialization with stratified organization most similar to unoperated controls. Fibrotic response was assessed by staining for α-SMA, an actin isoform incorporated into the contractile stress fibers of myofibroblasts that produce excess disorganized ECM and mediate stromal scarring.^55^ α-SMA-positive myofibroblasts were observed in the PBS-treated corneas, but not in the collagen gel or SIPN groups. Zonula occludens-1, a key constituent of the superficial epithelial tight junctions,^56^ was expressed in the SIPN and PBS-treated corneas, indicating re-epithelialization. Together, these data show that the SIPN enhances corneal restoration in an in vivo model of corneal stromal injury.

## Discussion

The personal and societal impact of vision loss and impairment affecting millions with corneal stromal injury can be devastating.^57^ Our armamentarium to tackle this challenge has been limited to corneal transplantation for a century,^58^ even as millions of patients lack access to donor tissue, surgical infrastructure, and the assiduous postoperative care required for successful keratoplasty. To this end, we have designed and characterized an in situ-forming SIPN stromal substitute composed of cross-linked collagen type I and physically entangled HA. This novel hydrogel has biomimetic mechanical and optical properties and promotes corneal regeneration in vivo following injury.

Our SIPN hydrogel was designed to recapitulate the native bidirectional interaction between keratocytes and the ECM. Collagen type I is the most abundant protein in the stromal ECM^23^ and provides essential keratocyte binding sites.^59^ Bovine collagen was selected for its clinical translatability because it is relatively low in cost and is widely available,^60^ it is less immunogenic than other sources^61^ and is already used in FDA-approved facial fillers.^62^ Glycosaminoglycans help to maintain the ordered spacing of stromal collagen fibers,^63^ critical for both the biomechanical properties and optical clarity of the cornea.^64^ HA has been shown to decrease epithelial matrix metallopeptidase 9 that can delay wound healing^65^ and attenuate transforming growth factor beta 1 signaling,^66^ which helps to drive stromal fibrosis.^67^ Collagen was cross-linked with bifunctional PEG using NHS ester chemistry, which facilitates wound adherence by reacting with primary amines in native stroma and is used in the FDA-approved tissue sealants.

To date, most hydrogel-based candidates under investigation for corneal replacement have been preformed implants.^68^^–^^73^ A handful of in situ-forming corneal substitutes, which can be injected to fill heterogenous defects, have been investigated. For example, the highly biocompatible and tunable gelatin methacryloyl (GelMA)-based gelatin for corneal regeneration (GelCORE) promoted wound healing in rabbits with lamellar injury.^21^ This approach requires photocross-linking for gelation, necessitating a light energy source. LiQD Cornea, composed of collagen-like peptide–PEG–fibrinogen, could gel in situ after cross-linking above 37°C and adhere to thrombin-coated corneal defects, performing similarly to syngeneic transplants in vivo and fostering regeneration in a feline model of both lamellar and full-thickness injury.^74^

In addition to the fact that it does not require light energy or a chemical initiator, the SIPN exhibits additional characteristics that make it a promising candidate for clinical use in the treatment of corneal wounds. Its mechanical properties are robust and tunable, potentially enabling fine control of structure for therapeutic applications, such as the delivery of growth factors or therapeutic cells. Increased HA concentration in the SIPN decreased its stiffness, possibly through increased interfibrillar spacing.^75^ This phenomenon may be advantageous for cell delivery because it has been shown that softer substrates inhibit myofibroblast differentiation^76^^,^^77^ and promote cellular stemness in the limbus.^78^ Moreover, the cornea exhibits viscoelastic behavior, which affects stromal cell behavior; we found that the SIPN's storage and loss moduli were similar to those of the native cornea.^79^^,^^80^ In addition, the hydrogel is highly transparent but its refractive index is lower than the native cornea owing to its higher water content. We do not believe this will be an issue clinically, because we utilize a contact lens after gel application, which not only protects the treated cornea but also can be used to correct refractive error during the early post-treatment period. Moreover, because the gel is designed to be re-modeled and replaced with stromal tissue and corneal crystallins over time, the reduced refractive power of the gel treatment is expected to be transient and not clinically significant in and of itself.^81^ Indeed, the SIPN further enhanced human CSSC viability over the collagen gel compared with physical collagen, indicating superior support for native recellularization. The SIPN was also able to maintain a more homogenous distribution of cells and prevent the gravity-mediated sedimentation of encapsulated CSSCs compared with controls. This finding suggests further usefulness in potentially augmenting the delivery of cells, another promising avenue for fostering corneal regeneration,^82^^–^^84^ where it is important to reliably deliver a predetermined number of cells without loss or inhomogeneity due to sedimentation. Importantly, application of the SIPN to injured corneas in vivo significantly decreased stromal defect size after 1 week compared with controls and supported ordered re-epithelialization most similar to uninjured corneas, with evidence of epithelial tight junction formation.

## Conclusions

We have developed a biocompatible SIPN of collagen type I and HA with tunable mechanical properties and excellent transparency and have demonstrated that it supports multilayered corneal epithelial regeneration in vivo after 1 week. The SIPN's constituents and cross-linking chemistry are used in FDA-approved products for therapeutic application, and its in situ-gelling dynamics are ideal for facile application in clinic and low-resource settings. Further studies are necessary to assess long-term wound healing characteristics, including long-term biological response and sustained optical clarity, to support progression to trials in patients suffering from corneal blindness due to stromal scarring.
